# Dysregulation of lipid metabolism in the liver affects ceramide content in the brain of male APP/PS1 mice

**DOI:** 10.1002/alz70855_096234

**Published:** 2025-12-23

**Authors:** Carlos Alfredo Silva‐Ramírez, Pablo Muriel, Claudia Perez‐Cruz

**Affiliations:** ^1^ CINVESTAV, Mexico City, DF, Mexico; ^2^ Cinvestav, Mexico City, DF, Mexico

## Abstract

**Background:**

Lipid accumulation in the liver leads to metabolic‐associated fatty liver disease (MAFLD), a pathology that has been associated with Alzheimer's disease (AD). MAFLD relates to alterations in lipid metabolism in several organs, including the brain. Impaired lipid metabolism in the brain leads to synthesizing toxic compounds, such as ceramides. Ceramide accumulation in the brain may lead to neuroinflammation. However, it is unknown if APP/PS1 mice develop alterations in lipid metabolism in the liver and whether this alteration may be associated with ceramide accumulation and neuroinflammation in the brain.

**Method:**

We determined the presence of neutral lipids and ceramides in the liver of six‐month‐old male APP/PS1 transgenic (TG) and their wild‐type (WT) littermate mice by histological staining and ELISA, respectively. We also determined the expression and localization of ceramides and glial fibrillary acidic protein (GFAP)+ astrocytes in the brain by immunofluorescence.

**Result:**

We observed higher ceramide content and microvesicular lipid accumulation in TG mice's hepatocytes compared to WT mice, an event accompanied by altered hepatic cytology. TG mice also showed an accumulation of ceramides in the somatosensory and entorhinal cortices compared to WT mice. Moreover, a higher number of GFAP+astrocyte with ceramide content was found in the *stratum radiatum* and entorhinal cortex of TG than WT mice.

**Conclusion:**

TG mice develop an altered lipid metabolism in the liver that may be associated with an enhanced accumulation of ceramides. Ceramides were highly expressed in GFAP+ astrocytes in TG mice, but not in WT controls. Our results add information about lipid metabolic alterations that may be associated with the onset of AD pathology, with special emphasis in the liver – brain axis.

